# Update: Interim Guidance for Health Care Professionals Evaluating and Caring for Patients with Suspected E-cigarette, or Vaping, Product Use–Associated Lung Injury and for Reducing the Risk for Rehospitalization and Death Following Hospital Discharge — United States, December 2019

**DOI:** 10.15585/mmwr.mm685152e2

**Published:** 2020-01-03

**Authors:** Mary E. Evans, Evelyn Twentyman, Eleanor S. Click, Alyson B. Goodman, David N. Weissman, Emily Kiernan, Susan Adkins Hocevar, Christina A. Mikosz, Melissa Danielson, Kayla N. Anderson, Sascha Ellington, Matthew J. Lozier, Lori A. Pollack, Dale A. Rose, Vikram Krishnasamy, Christopher M. Jones, Peter Briss, Brian A. King, Jennifer L. Wiltz, Lung Injury Response Clinical Task Force, Lung Injury Response Clinical Working Group, Lung Injury Response Clinical Task Force, Maleeka J. Glover, Paul C. Melstrom, Katherine R. Shealy, Stacy L. Thorne,, Lung Injury Response Clinical Working Group, Scott Aberegg, Carolyn S. Calfee, Sean J. Callahan, Annette Esper, Anne Griffiths, Dixie Harris, Don Hayes, Devika R. Rao, Lincoln S. Smith

**Affiliations:** ^1^National Center for Injury Prevention and Control, CDC; ^2^National Center for Chronic Disease Prevention and Health Promotion, CDC; ^3^Center for Global Health, CDC; ^4^National Institute for Occupational Safety and Health, CDC; ^5^Agency for Toxic Substances and Disease Registry, CDC; ^6^National Center for HIV/AIDS, Viral Hepatitis, STD, and TB Prevention, CDC; ^7^National Center on Birth Defects and Developmental Disabilities, CDC; ^8^National Center for Emerging and Zoonotic Infectious Diseases, CDC.; Office of the Deputy Director for Public Health Service and Implementation Science, CDC; National Center for Chronic Disease Prevention and Health Promotion, CDC; National Center for Chronic Disease Prevention and Health Promotion, CDC; National Center for HIV/AIDS, Viral Hepatitis, STD, and TB Prevention, CDC; University of Utah, Salt Lake City, Utah; University of California San Francisco Department of Medicine, San Francisco, California; University of Utah, Salt Lake City, Utah; Emory University, Atlanta, Georgia; Children’s Minnesota, Minneapolis, Minnesota; Intermountain Healthcare, Salt Lake City, Utah; Nationwide Children’s Hospital and The Ohio State University, Columbus, Ohio; University of Texas Southwestern Medical Center, Dallas, Texas; University of Washington and Seattle Children’s Hospital, Seattle; Washington.

*On December 20, 2019, this report was posted as an *MMWR* Early Release on the *MMWR* website (https://www.cdc.gov/mmwr).*

CDC, the Food and Drug Administration, state and local health departments, and public health and clinical stakeholders continue to investigate a nationwide outbreak of e-cigarette, or vaping, product use–associated lung injury (EVALI) (*1*–*4*). Clinical guidance from CDC and state partners for EVALI continues to evolve as more information about EVALI becomes available (*5*–*8*). Among EVALI patients who were rehospitalized or who died after discharge for an EVALI-related hospitalization, a recent study found a high rate of comorbidities and a median interval from discharge to readmission of 4 days and a median interval from discharge to death of 3 days; at least one quarter of rehospitalizations and deaths occurred within 2 days of discharge (*9*). The study findings prompted CDC, in consultation with the Lung Injury Response Clinical Working Group, to update guidance regarding timing of the initial postdischarge follow-up of hospitalized EVALI patients and other EVALI patient management. Updates to current clinical guidance include recommendations for discharge planning and optimized follow-up and case management after discharge that might reduce risk of rehospitalization and avert postdischarge mortality among patients hospitalized for EVALI. Specifically, guidance updates include 1) confirming no clinically significant fluctuations in vital signs for at least 24–48 hours before discharge; 2) ensuring outpatient primary care or pulmonary specialist follow-up, optimally within 48 hours of discharge (previously recommended within 2 weeks of discharge); 3) planning for discharge care, early follow-up, and management of any comorbidities; 4) arranging posthospitalization specialty care; 5) following best practices for medication adherence; and 6) ensuring social support and access to mental and behavioral health and substance use disorder services.

As of December 10, 2019, a total of 2,409 hospitalized EVALI cases have been reported to CDC, including 52 (2%) deaths among EVALI patients. Among 1,139 reported cases with patient hospital discharge by October 31, 2019, 31 (2.7%) patients were rehospitalized after initial discharge (median time to readmission: 4 days [interquartile range: 2–20 days]), and seven patients died following discharge after an EVALI hospitalization (median time to death: 3 days [interquartile range 2–13 days]) (*9*). Characteristics of EVALI patients who were rehospitalized or died following hospital discharge indicate that some chronic medical conditions, including cardiac disease, chronic pulmonary disease (e.g., chronic obstructive pulmonary disease and obstructive sleep apnea), and diabetes, and increasing age are risk factors leading to higher morbidity and mortality among some EVALI patients. For example, 70.6% of patients who were rehospitalized and 83.3 (five of six) of patients who died had one or more chronic conditions, compared with 25.6% of patients who were neither rehospitalized nor died (*9*). EVALI patients who were rehospitalized or died after discharge were older: the median ages of patients who died, were rehospitalized, and who neither died nor were rehospitalized were 54, 27, and 23 years, respectively (*9*).

Confirming stability of certain clinical parameters without clinically significant fluctuations in vital signs (Box) (Supplementary Figure, https://stacks.cdc.gov/view/cdc/83554) before discharge and careful hospital discharge and transition planning might help prevent rehospitalization or death, particularly among those patients with cardiac or chronic respiratory comorbidities who are at higher risk for rehospitalization or death (*9*). In addition, anxiety, depression, attention-deficit/hyperactivity disorder, and other mental or behavioral health conditions were common among all EVALI patients (*9*). Based on the high prevalence of these conditions, appropriate engagement with social and behavioral health services during care transition from hospital to the outpatient setting is also important.

BOX **Criteria for determining readiness for hospital discharge of patients with e-cigarette, or vaping, product use–associated lung injury (EVALI)**

- Patient is clinically stable for 24–48 hours before discharge
- Initial outpatient follow-up, optimally within 48 hours of discharge is scheduled
- Pulmonology follow-up within 2–4 weeks and at 1–2 months is scheduled
- Additional specialty outpatient follow-up is scheduled according to specific patient characteristics (e.g., endocrinology, cardiology, psychiatry, addiction medicine, physical therapy, pain medicine, and others as indicated)
- Discharge medication reconciliation and counseling of patient by inpatient pharmacist is completed
- Screening for mental health and substance use disorders and social needs and connection to appropriate social care (e.g., social work, behavioral health, community health) is established before discharge
- Health care providers have discussed e-cigarette, or vaping, cessation, documented patient quit plan, and offered evidence-based tobacco use cessation interventions, including behavioral counseling and medications

## Clinical Guidance Development

To develop this updated clinical guidance, CDC reviewed new data on rehospitalization and death after hospital discharge (*9*), and consulted with the Lung Injury Response Clinical Working Group regarding approaches to clinical management of suspected EVALI patients. Previous EVALI guidance has focused on 1) diagnosis (including obtaining an accurate history and conducting a physical examination that includes vital signs, pulmonary auscultation, and pulse oximetry; laboratory testing to rule out infectious etiologies; radiographic imaging; and consulting a specialist); 2) inpatient and outpatient management (including consideration of empiric administration of corticosteroids and antimicrobials, if indicated); 3) follow-up after hospital admission; and 4) considerations during the influenza season (Figure) (*5*,*7*). This updated guidance highlights health care system best practices for EVALI patients that might improve care quality and reduce the risk for adverse outcomes, including rehospitalization and death. Best practices include carefully assessing clinical readiness for discharge, comprehensive discharge planning (e.g., follow-up with specialty care providers), and ensuring follow-up by primary care or pulmonary specialist, optimally within 48 hours of hospital discharge.

**FIGURE:**
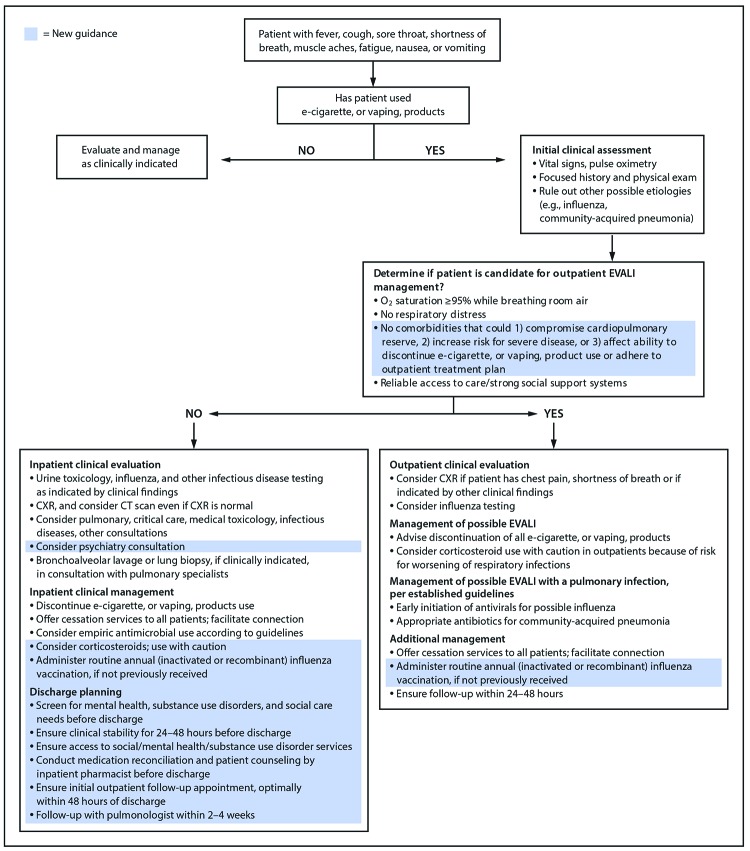
Updated algorithm for management of patients* with suspected e-cigarette, or vaping, product use–associated lung injury (EVALI), December 2019 * Influenza vaccination recommendations: https://www.cdc.gov/mmwr/volumes/68/rr/rr6803a1.htm?s_cid=rr6803a1_w.

## Updated Guidance: Discharge Planning

The occurrence of adverse clinical outcomes among EVALI patients shortly after hospital discharge (*9*) underscores the importance of ensuring that patients are clinically stable and have quality posthospital care transitions, which can improve patient outcomes (*10*).

**Assess clinical readiness for discharge.** Patients should be ready for discharge and meet discharge criteria for at least 24–48 hours before discharge, without clinically significant fluctuations in vital signs (Box) (Supplementary Figure, https://stacks.cdc.gov/view/cdc/83554).

**Assure social support and access to mental health and substance use disorder services.** Rehospitalized EVALI patients often continue to use e-cigarette, or vaping, products after initial hospitalization (Lung Injury Response Clinical Working Group, personal communication, December 2019). Therefore, during an inpatient admission and during outpatient follow-up, patients should be supported in their efforts to discontinue e-cigarette, or vaping, product use and should be educated that resuming use of e-cigarette, or vaping, products might result in recurrence of lung injury symptoms. EVALI patients might also benefit from evaluation for mental and behavioral health conditions by a social worker, behavioral health professional, psychologist or psychiatrist, or other member of the social care workforce to determine postdischarge support needs (*11*). The U.S. Department of Health and Human Services’ Substance Abuse and Mental Health Services Administration offers several helpful mental and behavioral health condition screening tools (*12*). In addition, tools such as the World Health Organization’s Alcohol, Smoking, and Substance Involvement Screening Test for adults (*12*) or the CRAFFT-N screening tools for adolescents (*13*) are available to help identify patient need for substance use treatment services (*14*). Approaches to changing behavior, including cognitive-behavioral therapy, contingency management, and motivational enhancement therapy, as well as multidimensional family therapy (a comprehensive family-centered treatment program) have been shown to be effective in reducing drug use in patients with cannabis use disorder, and addiction medicine services should be included in the care plan as appropriate (*15*,*16*). Evidence-based strategies are recommended for the treatment of tobacco product use and dependence (*17*). For patients aged <18 years who use e-cigarette, or vaping, products, health care professionals can consider the use of interventions that have been shown to increase cigarette smoking cessation among adults, including behavioral interventions (*18*). No medications are currently approved by the Food and Drug Administration for cessation of tobacco products, including e-cigarettes, in children and adolescents (*18*).

**Follow best practices for medication adherence.** A recent analysis found no significant difference in the percentage of discharged EVALI patients who received corticosteroid treatment while hospitalized among those who were rehospitalized, who later died, and who neither required rehospitalization nor died after discharge (*9*). However, clinicians working closely with CDC have reported that rehospitalized EVALI patients have at times not adhered to prescribed corticosteroid tapers (Lung Injury Response Clinical Working Group, personal communication, December 2019). Patient adherence to prescribed medications has been determined to be enhanced by inpatient pharmacist counseling before patient discharge (*19*,*20*) and that such counseling decreases rehospitalization. Thus, part of EVALI patient discharge planning should include inpatient pharmacist counseling, particularly for patients on a corticosteroid taper. Before hospital discharge, clinicians should evaluate EVALI patients for risk of secondary adrenal insufficiency (*21*) and other consequences of corticosteroid use (*22*) in the context of corticosteroid doses received and patient medical history; for patients who have had a prolonged corticosteroid course, clinicians should consider a corticosteroid taper and follow-up with an endocrinologist (*21*,*22*). Clinicians should also counsel patients about the signs and symptoms of adrenal insufficiency, such as fatigue, decreased appetite, gastrointestinal distress, myalgia, joint pain, salt craving, dizziness, and postural hypotension (*21*) and advise them to seek medical attention should these occur.

**Postdischarge medical follow-up.** Care transition and follow-up best practices include 1) scheduling follow-up appointments before hospital discharge and assigning patient navigators or community health workers to patients with significant barriers to care; 2) directly connecting patients to community services such as those addressing social determinants of health; 3) checking in by telephone or text; and 4) facilitating home visits by community health workers, home nursing services, or equivalent support staff for the most vulnerable patients (*23*,*24*).

**Initial outpatient follow-up.** Outpatient follow-up with primary care providers or pulmonology specialists within 48 hours after hospital discharge for EVALI might provide an opportunity to prevent adverse outcomes, including rehospitalization or death. Previous guidance recommended outpatient follow-up within 1–2 weeks (*5*–*8*); however, recent data support ensuring earlier follow-up, optimally within 48 hours (*9*). Early outpatient follow-up has been shown to be effective in improving management of other pulmonary conditions, including asthma (*19*). Outpatient follow-up with primary care providers or pulmonary specialists should include 1) clinically assessing for stable vital signs, physical exam, resolution or symptoms, and normalized laboratory tests; 2) continuing education about EVALI; 3) ensuring adherence with medication regimens such as tapering of corticosteroids (if prescribed at the time of hospital discharge); 4) reinforcing the importance of abstinence from e-cigarette, or vaping, product use; 5) facilitating connection to outpatient care by all providers or services indicated by patients’ medical history or conditions; 6) connecting patients to needed social, mental health, and substance use disorder resources; and 7) establishing connection to necessary services.

**Pulmonary specialist follow-up.** Longer-term pulmonary follow-up should generally occur within 2–4 weeks after discharge (often at completion of the corticosteroid taper) to assess pulmonary function and resolution of radiographic findings (Lung Injury Clinical Working Group, personal communication, December 2019). In addition to this new guidance, CDC continues to recommend follow-up testing 1–2 months after discharge, which might include spirometry, diffusing capacity of the lung for carbon monoxide, and chest x-ray (*7*,*8*).

**Other follow-up.** Patients who have experienced prolonged immobilization during hospitalization (particularly those with intensive care unit–related deconditioning and muscle atrophy) might benefit from physical therapy. Ongoing engagement with addiction medicine and mental health services should be considered.

New data have provided insight into characteristics of EVALI patients who have been rehospitalized or have died after an EVALI-related hospitalization. In consultation with the Lung Injury Response Clinical Working Group, CDC is using these data to update clinical guidance to include recommendations for outpatient follow-up, optimally within 48 hours after hospital discharge and for specific considerations concerning discharge planning and care transitions. Incorporating these updated recommendations into the management of patients with EVALI might reduce their risk for rehospitalization and avert further mortality among patients hospitalized for EVALI.

**Summary**

**What is already known about this topic?**

In a recent examination of rehospitalization and death among previously hospitalized patients with e-cigarette or vaping, product use–associated lung injury (EVALI), at least one quarter of rehospitalizations and deaths occurred within 2 days of discharge; comorbidities were common among patients who were rehospitalized or who died after discharge.

**What is added by this report?**

Updated guidance recommends posthospitalization outpatient follow-up, optimally within 48 hours of discharge, and emphasizes the importance of preparation for hospital discharge and postdischarge care coordination to reduce risk of rehospitalization and death among hospitalized EVALI patients.

**What are the implications for public health practice?**

Incorporating this updated guidance into the management of hospitalized EVALI patients might reduce EVALI-associated morbidity and mortality.
